# Work Expectations and Trajectories of Depressive Symptoms and Passive Suicidal Ideation Among Baby Boomers

**DOI:** 10.1093/geroni/igab046.2130

**Published:** 2021-12-17

**Authors:** Linh Dang, Briana Mezuk

**Affiliations:** 1 University of Michigan School of Public Health, Ann Arbor, Michigan, United States; 2 University of Michigan, Ann Arbor, Michigan, United States

## Abstract

Expectations regarding work (e.g., probability of retiring at a certain age), whether realized or not, may influence mental health, however there is limited quantitative research on this question. This study examined the longitudinal relationship between expectations of full-time work after age 62 and depressive symptoms and passive suicidal ideation among Baby Boomers, a generation that experienced the Great Recession as they neared retirement. Data came from the Health and Retirement Study, 2008 - 2016 (N = 8,954, mean age = 55.3, 52.2% female, 77.8% non-Hispanic White). Clinically-relevant depressive symptoms were indexed by the Composite International Diagnostic Interview (CIDI). Expectation (probability) of working after age 62 was modeled continuously (range: 0 to 1). Multivariate mixed-effects logistic regression models of screening positive on the CIDI and passive suicide ideation were fit, separately, adjusting for demographics, household income and wealth, and health characteristics. Respondents working at baseline were less likely to screen positive on the CIDI longitudinally (OR: 0.36, 95% CI: 0.26 - 0.51), and while expectations were inversely associated with screening positive on the CIDI this was not significant after accounting for work status (OR: 0.68, 95% CI: 0.43 - 1.09, p=0.104). Longitudinally, higher expectations of working were inversely associated with passive suicidal ideation (OR: 0.54, 95% CI: 0.32 - 0.92) even after accounting for working status. Future research will examine variation in these relationships by contextual factors like wealth, sex, and race/ethnicity to clarify how these features shape the association between work and mental health for this generation of older adults.

